# Digital and Non-Digital Solidarity between Older Parents and Their Middle-Aged Children: Associations with Mental Health during the COVID-19 Pandemic

**DOI:** 10.3390/ijerph191912560

**Published:** 2022-10-01

**Authors:** Woosang Hwang, Xiaoyu Fu, Maria Teresa Brown, Merril Silverstein

**Affiliations:** 1 Department of Human Development and Family Sciences, Texas Tech University, Lubbock, TX 79409, USA; 2 Department of Human Development and Family Science, Syracuse University, Syracuse, NY 13244, USA; 3 School of Social Work, Syracuse University, Syracuse, NY 13244, USA; 4 Aging Studies Institute, Syracuse University, Syracuse, NY 13244, USA; 5 Department of Sociology, Syracuse University, Syracuse, NY 13244, USA

**Keywords:** intergenerational solidarity, intergenerational digital communication, digital solidarity, mental health, COVID-19 pandemic

## Abstract

We incorporated intergenerational digital communication (frequency of texting, video call, and social media interaction) into the intergenerational solidarity paradigm and identified new types of intergenerational and digital solidarity with adult children among older parents during the COVID-19 pandemic. In addition, we examined whether those types are associated with older parents’ mental health (depressive symptoms, psychological well-being, and self-esteem). We used the 2021/2022 wave of the Longitudinal Study of Generations (LSOG), and a sample of 519 older parents (mean age = 69 years). Latent class analysis identified four classes describing intergenerational and digital solidarity with adult children (*distant-but-digitally connected*, *tight-knit-traditional*, *detached*, and *ambivalent*). We found that older parents who had *distant-but-digitally connected* and *tight-knit-traditional* relationships with their adult children reported better mental health, compared to those who had *detached* and *ambivalent* relationships with their adult children during the COVID-19 pandemic. Our findings suggest that intergenerational digital communication should be considered as a digital solidarity in intergenerational solidarity paradigm, which is useful for measuring multidimension of intergenerational relationships within family members during and after the pandemic.

## 1. Introduction

Lockdown restrictions and social distancing measures in response to the COVID-19 pandemic have resulted in dramatic reductions in social interaction between family members, increasing the risk of social isolation and worsening mental health among older adults [1,2]. Psychological distress and loneliness are higher than usual at older ages during the pandemic [3,4,5]. Being a major part of older adults’ social systems, intergenerational relationships are primary sources of social contact and support to alleviate depression or isolation during the outbreak of COVID-19 [6,7].

However, with limited face-to-face contact, family members may turn to digital forms of communication to strengthen social integration between generations during the pandemic [8]. Many studies focused on the types of intergenerational contact and their associations with older adults’ well-being [9,10]. However, older parents and adult children are interconnected in various dimensions of intergenerational relationships simultaneously [11,12]. Changes in the form of contact are associated with changes in other domains of relationships. It is difficult to understand the impact of intergenerational relationships on older parents’ mental health without considering the interrelationships among different dimensions of intergenerational relationships. As a result, little is known about how multiple dimensions of intergenerational ties combined with digital communication affect older adults’ mental health during the pandemic. To fill this gap, this study developed a typology of intergenerational and digital solidarity to examine the different patterns of the multifaceted parent–child relationships during the pandemic and the association between types of parent–child solidarity and older parents’ mental health.

## 2. Literature Review

### 2.1. Intergenerational Solidarity

Intergenerational solidarity is defined as the social cohesion among family members across generations [11]. As a systematic scheme to organize and understand the interrelated domains in intergenerational relationships, the intergenerational solidarity paradigm was initially composed of six dimensions: affection (emotional closeness), association (frequency of interaction), structure (geographic proximity), consensus (agreement on values), filial norms (familial obligations), and function (exchange of support and assistance) [11]. While intergenerational solidarity was originally developed to depict the positive interactions in intergenerational relations, the dimension of conflict was added to the framework in later refinements to account for both the positive and negative aspects of these relationships [13]. Instead of being the opposite of affection, conflict can simultaneously occur with affection in interpersonal relationships. Such contradiction experienced in family relations due to the coexistence of positive and negative emotions is described as ambivalence [14]. The solidarity-conflict model thus was adapted to capture both the negative aspects and the ambivalence in family life.

In accordance with the theory, empirical studies have shown that intergenerational solidarity is a multidimensional construct. Researchers have identified different types of intergenerational relations based on the dimensions of relations examined in clustering analysis. Studies that examined positive attributes of solidarity have revealed five intergenerational solidarity types: tight-knit, sociable, intimate but distant, obligatory, and detached [12,15]. Studies focusing on the affection and conflict dimensions have identified four types of intergenerational solidarity across cultures: amicable, detached, ambivalent, and disharmonious [16,17]. When investigating a larger range of solidarity attributes and conflict, Van Gaalen and Dykstra [18] classified intergenerational relations into five types: harmonious, ambivalent, obligatory, affective, and discordant. Although studies vary in the selected domains examined in their analyses, only a few typology studies, have examined associational solidarity [12,16,17,19]. Even more rare are studies examining digital communications as a complement to in-person and telephone contact [18,20,21].

### 2.2. Digital Solidarity in Intergenerational Relationships

Digital solidarity is proposed as an extended dimension of associational solidarity to acknowledge the role of digital forms of communication in maintaining intergenerational solidarity [22]. With the widespread use of smartphones, digital communication use is growing among older generations. For example, in 2021, 73% of adults ages 50–64 reported using social media sites, and 45% of adults ages 65 and older reported doing so [23]. The advance of communication technology is linked to increased frequency of interaction with family members of another generation. In data collected from 2008 to 2010, only 31.1% of surveyed mothers reported instant messaging or emailing their adult children in the previous year, whereas in 2016, 95.4% of mothers reported texting or emailing their adult children in the previous year [22]. Digital communication has known impacts on other dimensions of solidarity. Shifts in digital communication are associated with corresponding changes in-person communication and helping behavior in intergenerational relationships [24]. Further, parents and adult children can exchange expressive support promptly by virtue of the immediacy of digital communication [25]. Therefore, digital communication across generations represents a novel and possibly independent domain of intergenerational solidarity and requires further investigation.

Digital communication has played a vital role in family members staying connected and supported during the COVID-19 pandemic. The main consequence of the policy measures to contain the COVID-19 pandemic was the reduction in physical contact and lack of social support, which were detrimental to older adults’ mental health [2]. Because of older adults’ higher risks of COVID-19 infections and severe illness [26], younger generations tended to reduce their face-to-face contact with older parents. Digital technologies provided various options for intergenerational communication that required no physical contact, less time investment, and lower or no cost, such as FaceTime, Messenger, and Skype [27]. The virtuality of digital communication made it exceptionally valuable for older parents and adult children to stay connected during the COVID-19 pandemic. Using a national panel sample of adults older than 50 years, one study found an increase in physical and social isolation, but no change in digital isolation, when the social distancing policy was implemented [28]. About half of older adults increased digital communication (i.e., video calls, instant messages, and social media) during COVID lockdowns, especially among those who reduced face-to-face contact [8]. Therefore, digital communication enables older parents, who are geographically distant from adult children or have insufficient face-to-face contact, to stay connected.

### 2.3. Intergenerational and Digital Solidarity and Older Adults’ Mental Heath

The role of intergenerational solidarity in older adults’ mental health has been examined extensively in previous decades [29,30]. Existing literature assessing the well-being of older adults has focused on one dimension of intergenerational relationships [29,31,32] or typologies of intergenerational relationships based on multiple dimensions [33]. Older adults in the tight-knit type of parent–child relationship reported the lowest depressive symptoms, while those in the ambivalent type reported the highest depressive symptoms [34]. In the context of the COVID-19 pandemic, it was not clear whether the association between typologies of intergenerational solidarity and older adults’ mental health would be impacted by the reduction in face-to-face contact.

Although digital communication has been recommended as a strategy to combat social isolation and mental illness caused by COVID-19 [35], empirical studies have yielded mixed findings regarding the association between digital communication and older adults’ mental health (Teo et al., 2015). Some studies found that older adults who reported increased or unchanged frequency of digital contact with non-coresident family members were less likely to experience increased depressive symptoms compared to those who reported decreased digital contact [3,10]. Other research addressed the positive emotions associated with more frequent contact with family members using phone or email for older adults in long-term care facilities [7]. Using longitudinal data, digital communication was found to be negatively associated with loneliness among older adults through the mechanism of social support and social contact [36,37]. However, other studies found that digital forms of communication were associated with negative effects on older adults’ mental health [38], especially for those who lived alone [39]. Some studies have shown that digital contact is not a high-quality alternative to in-person contact, given the unique benefits of face-to-face contact with family members to older adults’ general mental health during the outbreak [9,40]. These previous studies have focused on the frequency of physical and digital contact during the pandemic, but little attention has been paid to the other dimensions of solidarity that interrelate with various forms of contact in parent and adult-child relationships. Thus, in the current study, we examine overall patterns of intergenerational and digital solidarity and traditional non-digital dimensions of solidarity, and their associations with older adults’ mental health.

### 2.4. The Current Study

The first aim of this study was to integrate digital communication with legacy dimensions of intergenerational solidarity to identify types of relationships with adult children as reported by older parents during the COVID-19 pandemic. We included affectual, consensual, associational (in-person and phone contact), structural, and conflict dimensions of solidarity, and added digital solidarity (texting, video call, and social media interaction). We anticipated that a *distant-but-digitally connected* type would emerge in the data alongside other types such as types that are strongly connected on all dimensions of solidarity and weakly connected on all dimensions of solidarity. The second aim was to determine whether having digitally connected relationships with children was associated with older parents’ mental health (depressive symptoms, psychological well-being, and self-esteem). We hypothesized that older parents who had strong solidarity with their adult children on all dimensions during the pandemic would report better mental health, compared to those with weak solidarity with their adult children, but that digital communication would compensate for low amounts of more traditional forms of contact.

## 3. Methods

### 3.1. Sample

This study used data from the Longitudinal Study of Generations (LSOG), a multigenerational and multi-panel study of 3681 individuals within 418 families. The LSOG began in 1971 (Wave-1) as a cross-sectional study. Three-generation families including grandparents (G1), parents (G2), and grandchildren (G3) were recruited from a large health maintenance organization in southern California. The LSOG became the longitudinal study in 1985 (Wave-2) and continued to collect data from respondents up to 2022 for a total of ten waves of measurement. In addition, great-grandchildren (G4) were added to the sample on a rolling basis beginning in the 1991 study (Wave-4) when they reached the age of 16. Data were initially collected solely by mail-back paper questionnaire and the web-survey option was added beginning with the 2005 study (Wave-8) (for more details, see Bengtson, 2013).

Given that this investigation focused on older adults’ intergenerational and digital solidarity with middle-aged children and their well-being during the COVID-19 pandemic, we used data from the G3 generation in Wave-10 (November 2021 to April 2022). Among the 618 G3s who participated in Wave-10, we derived a subsample of 553 G3s and relied on their reports of intergenerational solidarity with their oldest child because firstborns are, on average, more intensively involved with their parents than are other children [41,42]. Given that our interest was in older parents who lived apart from their adult children, we excluded 34 G3s who lived with their oldest child. The final sample consisted of 519 G3s with a mean age of 69 years.

### 3.2. Measures

**Dependent Variables.** Analyses assessed three aspects of mental health: depressive symptoms, psychological well-being, and self-esteem. Depressive symptoms were measured by eight negative affect items (e.g., “I was bothered by things that do not usually bother me”) and two positive affect items (e.g., “I enjoyed life”) from the Center of Epidemiologic Studies Depression (CES-D) short form [43,44]. Response options ranged from (1) *rarely or none of the time* to (4) *most or all of the time.* After reverse coding the two positively worded items, we used a mean score of ten items, with higher scores indicating more depressive symptoms (Cronbach’s alpha = 0.80).

Psychological well-being was measured by five positive affect items (e.g., “Did you ever feel pleased about having accomplished something?”) and five negative items (e.g., “Did you ever feel upset because someone criticized you?) from the Bradburn Scale of Psychological Well-Being [45]. Response options were (0) *no* or (1) *yes*. After reverse coding the negative affect items, we summed all items (range: 0–10), with higher scores indicating better psychological well-being (Cronbach’s alpha = 0.65).

Self-esteem was measured by two negatively worded items (e.g., “All in all, I am inclined to feel that I am a failure”) and two positively worded items (e.g., “On the whole, I am satisfied with myself”) from the Rosenberg Self-Esteem Scale [46]. Response options ranged from (1) *strongly disagree* to (4) *strongly agree*. After reverse coding the two negatively worded items, we used a mean score of four items, with higher scores indicating greater self-esteem (Cronbach’s alpha = 0.75).

**Independent Variables.** The LSOG collected data on six dimensions of intergenerational solidarity—affectual, consensual, associational, digital, structural, and conflict—with adult children. Affectual solidarity with a child was measured by one item: “Taking everything into consideration, how close do you feel the relationship is between you and your child at this point in your life?” Responses ranged from (1) *not at all close* to (6) *extremely close*. Consensual solidarity with a child was measured by one item: “How similar are your opinions and values about life to those of your child at this point in time?” Responses ranged from (1) *not at all similar* to (6) *extremely similar*. Associational solidarity was measured by two separate items for frequency of in-person contact and frequency of audio telephone contact with a child. Responses ranged from (1) *not at all* to (6) *daily or more often*. Digital solidarity was measured by three separate items for frequency of texting, video calls, and social media interaction with a child. Responses ranged from (1) *not at all* to (6) *daily or more often*. Structural solidarity with a child was measured by one item regarding geographical proximity. Responses ranged from (1) *more than 500 miles from me* to (6) *less than 5 miles*. Conflict with a child was measured by one item: “Taking everything into consideration, how much conflict, tension, or disagreement do you feel there is between you and your child at this point in your life?” Responses ranged from (1) *not at all* to (6) *a great deal*.

**Control variables.** We included controls for G3s’ age, gender (0 = *male*, 1 = *female*), race (0 = *others*, 1 = *white*), education (0 = *did not finish high school*, 6 = *doctoral degree*), marital status (0 = *others*, 1 = *married or cohabiting*), and annual income (1 = *less than $20,000*, 11 = *$200,000 and more*). G3s’ health status was measured by three binary items about the presence of a serious chronic health condition (cardiovascular diseases, cancer, diabetes, and Alzheimer’s disease). We converted these measures to a dichotomous variable (0 = *no chronic condition*, 1 = at least one chronic condition). We also included controls for children’s age, gender (0 = *male*, 1 = *female*), and biological/stepparent–child relationship (0 = *step relationship*, 1 = *biological or adoptive relationship*).

### 3.3. Analytic Strategy

To develop a typology of solidarity from its constituent dimensions, we used latent class analysis, a person-centered approach that identifies unobserved subgroups from combinations of observed indicators based on respondents’ reports [47]. We employed a BCH three-step latent class analysis, a bias-adjusted method that corrects classification errors [48]. Given that intergenerational solidarity measures were originally comprised of ordinally scaled items, we initially conducted a latent profile analysis. However, we were not able to identify the best-fitting model because optimal latent classes were not well-differentiated. Therefore, we dichotomized intergenerational solidarity indicators and employed latent class analysis. Based on a six-point scale, each solidarity indicator (except for the three noted below) was dichotomized into low (range from 1 to 3) and high (range from 4 to 6). Given that video call, social media interaction, and conflict were strongly negatively skewed, they were dichotomized using a different cut-off (low = 1 to 2; high = 3 to 6) (for more details, see Table 1).

In the first step, we employed a latent class analysis using the nine dichotomized intergenerational solidarity indicators. We selected the optimal number of latent classes using three criteria: the Bayesian Information Criterion (BIC), the Consistent Akaike Information Criterion (CAIC), and entropy. The latent classes with the smallest BIC and CAIC, and entropy values over 0.8 were chosen as the best-fitting model [47]. In the second step, older adults were assigned a probability of intergenerational solidarity membership in each latent class. In the third step, analyses of associations between intergenerational solidarity class membership and covariates with mental health were conducted, with analyses weighted by latent class probability membership. We used full-information maximum likelihood estimation to account for missing values [49].

## 4. Results

### 4.1. Results of Descriptive Analysis

Results of descriptive analysis of older adults’ demographic characteristics and study variables are presented in Table 1. Most participants in the sample were white and married or cohabiting. Children’s mean age was 43 years and 52% were sons and 48% daughters. In terms of intergenerational solidarity with children, the measures for video calls, social media interaction, and conflict were skewed toward lower values and measures for affection, consensus and texting skewed toward higher values.

### 4.2. Results of Latent Class Analysis

**Figure 1:**
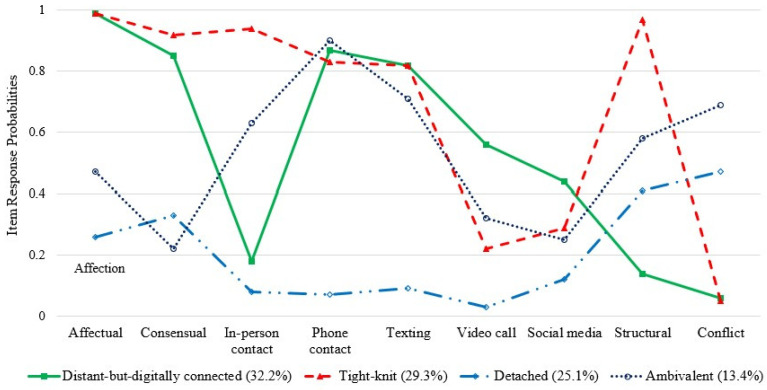
Item response and latent class probabilities in four international solidarity with adult children classes.

Latent class analysis statistics and fit indices are displayed in Table 2. Two fit indices (BIC and CAIC) and entropy values indicated that a four-class model was the optimal fitting model. Item response and latent class probabilities of the four-class model are displayed in Figure 1. We identified the following types of intergenerational solidarity with adult children: (1) *distant-but-digitally connected* (affection, consensual, phone contact, texting, video call, and social media interaction were high but proximity, in-person contact, and conflict were low; 32.2%), (2) *tight-knit-traditional* (all item response probabilities were high except video call, social media interaction, and conflict; 29.3%), (3) *detached* (all item response probabilities except conflict were low; 25.1%), and (4) *ambivalent* (affection, in-person contact, phone contact, and conflict were high; 13.4%).

### 4.3. Associations between Intergenerational Solidarity Latent Classes and Mental Health

We employed multivariate regressions weighted by probabilities of latent class membership to determine how membership in different classes of intergenerational solidarity with adult children (reference group: *detached*) was associated with three measures of older parents’ mental health. Results are presented in Table 3. We found that older parents who had *distant-but-digitally connected* and *tight-knit-traditional* relationships with adult children reported fewer depressive symptoms and better psychological well-being and self-esteem, compared to those who had a *detached* relationship with adult children. Older parents who had an *ambivalent* relationship with adult children reported higher depressive symptoms than those who had a *detached* relationship with adult children. Additionally, we employed a multi-group contrast of mean values of mental health in four groups (Table 4). We found that older parents who had *distant-but-digitally connected* and *tight-knit-traditional* relationships with adult children reported fewer depressive symptoms and better psychological well-being and self-esteem, compared to those who had an *ambivalent* relationship with adult children. In addition, these three aspects of mental health were not significantly different between older parents in the *distant-but-digitally connected* and *tight-knit-traditional* latent classes.

## 5. Discussion

Our results suggest that digital communication (i.e., video call, instant messaging, and social media) enabled older and younger generations to maintain contact as a substitute for in-person visits during the COVID-19 pandemic. Historically, we know little about how the use of digital communication created new forms of intergenerational solidarity between generations, or whether these new forms were associated with mental health. In this study, we aimed to provide an updated intergenerational solidarity theoretical framework, including digital communication, and examined the association of this new framework with mental health of older parents. That our data were collected in 2021–2022 during the COVID-19 pandemic (albeit in its less acute period) provided the opportunity to examine these issues when in-person contact carried some risk and families were under stress.

The first hypothesis, that we would identify distinct latent classes of intergenerational solidarity with adult children among older parents, was confirmed in this sample. We identified four distinct intergenerational solidarity latent classes: *distant-but-digitally connected*, *tight-knit-traditional*, *detached*, and *ambivalent*. The identification of the *distant-but-digitally connected* latent class among older parents extends the intergenerational solidarity model by demonstrating how digital communication was integrated into other dimensions of the intergenerational solidarity model during the pandemic. As we expected, the main difference between *distant-but-digitally connected* and *tight-knit-traditional* latent classes were geographical proximity and frequency of in-person and digital contact between older parents and their adult children. We found that older parents who lived far away from their adult children had more video call and social media interaction with adult children during the pandemic compared to those who lived close to their adult children. Consequently, our finding can be interpreted that video call and social medial interaction with adult children replaced in-person contact with adult children because of geographic distance and social distancing during the pandemic. Interestingly, the frequency of texting was similar among older parents who were in the *distant-but-digitally connected* and *tight-knit-traditional* latent classes. Given that the use of smartphones has become more common across all generations, texting including instant messaging can be seen as a widely used digital communication between older parents and adult children regardless of physical distance and to some degree substitutes for voice-phone use.

We also identified an *ambivalent* latent class in older parents’ relationships with their oldest child. Parents in this class were also emotionally closer with their children than would be expected, a finding supporting previous studies that positive forms of solidarity and conflict can co-occur with conflict in intergenerational relationships—a condition often referred to as ambivalence [13,21]. We found that older parents in this class also reported low consensual solidarity with adult children. For this reason, we speculate that high conflict might stem from differences in values and opinions between older parents and adult children.

We found that older adults in *distant-but-digitally connected* and *tight-knit-traditional* latent classes reported better mental health during the pandemic compared to those who were in *detached* and *ambivalent* latent classes. This finding confirms our second hypothesis that older parents who perceived strong solidarity with and without a digital element (*distant-but-digitally connected* and *tight-knit-traditional*) with their adult children would report better psychological well-being compared to those who perceived weak solidarity (*detached* and *ambivalent*) with their adult children. As we mentioned, the main difference between the *distant-but-digitally connected* and *tight-knit-traditional* classes is the level of digital communication between older parents and adult children. However, older adults’ mental health was not significantly different between these two latent classes. Therefore, our finding provides some evidence that digital solidarity would be beneficial for older parents’ psychological well-being by compensating for low in-person contact.

Our finding should be carefully interpreted because of several limitations. First, the analysis did not consider functional solidarity with adult children (receiving support from adult children and providing support to adult children) because the LSOG measured exchange of support with all children rather than exchange of support with a specific child. Second, it is possible that digital solidarity could be influenced by both older parents’ and adult children’s socioeconomic status. For example, highly educated older parents and adult children are more likely to communicate digitally with each other than those who with lower education levels. Therefore, we recommend that future studies examine whether intergenerational digital communication can vary according to both older parents’ and adult children’s socioeconomic conditions using dyadic data analysis. Third, our analyses were based on cross-sectional data. Thus, we were not able to explore the relationship between intergenerational solidarity classes and older parents’ mental health over time to strengthen causal attribution of the results. Fourth, the original LSOG sample overrepresented white and middle-income families and underrepresented minority and low-income families. In addition, the LSOG began as a regional study in Southern California. Therefore, we recommend caution when generalizing our results.

Nevertheless, the present study is theoretically and methodologically innovative, advancing our understanding of how traditional and digital forms of solidarity with adult children are associated with their psychological well-being during a unique period when constraints imposed by pandemic highlighted the importance of alternative strategies older parents use to maintain intergenerational solidarity. The findings from this study reveal several applications of intergenerational solidarity for programmatic interventions, policy development, and clinical research. First, programs that foster intergenerational integration have relied on the intergenerational solidarity paradigm in their development and assessment. A recent review explicitly used the dimensions of intergenerational solidarity as a unifying framework to provide a meta-review of 31 programs in 11 countries designed to strengthen intergenerational solidarity [50]. Therefore, the integration of digital solidarity into the intergenerational solidarity paradigm will encourage researchers and practitioners to include digital communication when evaluating multidimensional solidarity between generations in the digital age.

Second, as a guiding principle for policy formation, intergenerational interdependence is often stressed as a means for achieving a humane society for older and younger people. Seedsman [51] writes that “the notion of intergenerational solidarity is held to be the social glue that offers the opportunity for families, communities, and society to work together to create a stable and supportive environment across all generations” (p. 215). Policymakers have keen interest in how families serve the needs of their older relatives through intergenerational family solidarity. Therefore, the intergenerational and digital solidarity paradigm would be informative for developing policies that promote healthy and active aging.

Third, regarding the COVID-19 pandemic, gerontologists and geriatricians are rightfully concerned about the consequent social isolation experienced by many older adults. Enhancing social and family connectivity through intergenerational digital engagement, as well as through more conventional means, is viewed as a highly promising intervention to reduce loneliness and activate support networks among older people [52]. When evaluating an intergenerational mentoring program, Lee and Kim [53] found that technology mentoring between older adults and youth increased e-health literacy and reduced social isolation among older participants. Based on our findings, we suggest that digital communication within families has the potential to strengthen the delivery of informal care by adult children as medical partners. For the above reasons and more, we suggest that greater attention be devoted to strengthening the ability of older adults to adapt to the challenges imposed by the COVID-19 pandemic in maintaining intergenerational ties, and, by extension, to the relentless pace of technological innovation which predated and will postdate this once-in-a-hundred-year health crisis.

## Figures and Tables

**Table 1:** Results of Descriptive Analysis regarding Demographic and Study Variables.

Variables		Older Adults (*n* = 519)	
	*Range*	*M* (*SD*)	*n* (%)
Demographic Variables			
Age		69.07 (3.78)	
Gender			
Male			219 (42.2)
Female			297 (57.2)
Race			
White			497 (95.8)
Other ethnic groups			22 (4.2)
Education	1–6	3.58 (1.28)	
Marital status			
Married or cohabitating			410 (79.0)
Others			108 (20.8)
Annual income	1–11	5.68 (3.03)	
Health status			
Healthy			400 (77.1)
Unhealthy			119 (22.9)
Children’s age		43.27 (7.22)	
Children’s gender			
Son			270 (52.0)
Daughter			247 (47.6)
Parent–child relations			
Biological or adoptive children			442 (85.2)
Stepchildren			76 (14.6)
Number of children		2.75 (1.47)	
Intergenerational Solidarity			
Affectual solidarity	1–6	4.26 (1.48)	
Low group (range 1–3)			134 (25.8)
High group (range 4–6)			385 (74.2)
Consensual solidarity	1–6	3.97 (1.31)	
Low group (range 1–3)			169 (32.6)
High group (range 4–6)			341 (65.7)
Associational solidarity: In-person contact	1–6	3.32 (1.38)	
Low group (range 1–3)			289 (55.7)
High group (range 4–6)			228 (43.9)
Associational solidarity: Phone contact	1–6	3.91 (1.41)	
Low group (range 1–3)			173 (33.3)
High group (range 4–6)			344 (66.3)
Digital solidarity: Texting	1–6	3.88 (1.66)	
Low group (range 1–3)			194 (37.4)
High group (range 4–6)			324 (62.4)
Digital solidarity: Video call	1–6	2.05 (1.56) ^a^	
Low group (range 1–2)			361 (69.6)
High group (range 3–6)			155 (29.9)
Intergenerational Solidarity			
Digital solidarity: Social media	1–6	1.96 (1.34) ^a^	
Low group (range 1–2)			363 (69.9)
High group (range 3–6)			152 (29.3)
Structural solidarity	1–6	3.29 (1.94)	
Low group (range 1–3)			251 (48.4)
High group (range 4–6)			267 (51.4)
Conflict	1–6	2.02 (1.30) ^a^	
Low group (range 1–2)			388 (74.8)
High group (range 3–6)			128 (24.7)
Mental Health			
Depressive symptoms	1–4	1.59 (0.44)	
Psychological well-being	0–10	7.46 (1.98)	
Self-esteem	1–4	3.43 (0.46)	

Note: ^a^ = the absolute value of skewness exceeds 1.

**Table 2:** Latent Class Analysis Statistics and Fit Indices.

Model	Classes (*n*)	BIC	CAIC	Entropy
Model 1	1	5902.04	5911.04	-
Model 2	2	5386.61	5405.61	0.80
Model 3	3	5255.24	5284.24	0.81
**Model 4**	**4**	**5236.76**	**5275.76**	0.80
Model 5	5	5249.59	5298.59	0.83
Model 6	6	5276.17	5335.17	0.82

Note: Bolded values indicate best fit for each respective statistic. BIC = Bayesian Information Criterion. CAIC = Consistent Akaike Information Criterion.

**Table 3:** Associations of Class Memberships with Mental Health.

Variables	Older Adults (*n* = 519)		
	Depressive Symptoms	Psychological Well-Being	Self-Esteem
	*b* (*SE*)	*b* (*SE*)	*b* (*SE*)
Class Memberships (ref: Detached)			
Distant-but-digitally connected	−0.12 (0.06) ^†^	0.65 (0.28) *	0.26 (0.06) ***
Tight-knit-traditional	−0.11 (0.06) ^†^	0.73 (0.27) **	0.24 (0.06) ***
Ambivalent	0.17 (0.08) *	−0.59 (0.36)	0.04 (0.08)
Covariates			
Age	−0.00 (0.05)	0.02 (0.02)	−0.00 (0.00)
Female (ref: male)	0.02 (0.03)	−0.22 (0.17)	−0.06 (0.04)
White (ref: others)	−0.02 (0.08)	−0.93 (0.33) **	−0.09 (0.07)
Education	−0.01 (0.01)	0.01 (0.07)	0.00 (0.01)
Married/cohabiting (ref: others)	−0.16 (0.05) **	0.59 (0.23) *	0.11 (0.05) *
Annual income	−0.01 (0.00)	0.04 (0.03)	0.01 (0.00) *
Healthy (ref: unhealthy)	0.08 (0.04)	−0.06 (0.20)	−0.04 (0.04)
Children’s age	0.00 (0.00)	−0.00 (0.01)	−0.00 (0.00)
Daughters (ref: sons)	−0.00 (0.03)	−0.14 (0.17)	0.00 (0.03)
Biological/adoptive children (ref: stepchildren)	0.14 (0.05) *	−0.77 (0.26) **	−0.15 (0.06) *

Note: ref. = reference group. ^†^ *p* < 0.10. * *p* < 0.05. ** *p* < 0.01. *** *p* < 0.001.

**Table 4:** Results of Paired Comparison Test of Mental Health across Four Intergenerational Solidarity Latent Classes.

	Class 1 Distant-But-Digitally Connected	Class 2 Tight-Knit-Traditional	Class 3 Detached	Class 4 Ambivalent	
Mental health	*M* (*SE*)	*M* (*SD*)	*M* (*SD*)	*M* (*SD*)	*p*-value
Depressive symptoms	1.52 (0.03)	1.53 (0.03)	1.64 (0.05)	1.82 (0.06)	4 > 1 & 2 *** 4 > 3 * 3 > 1 & 2 ^†^
Psychological well-being	7.77 (0.15)	7.85 (0.15)	7.12 (0.22)	6.52 (0.28)	1 & 2 > 4 *** 1 > 3 * 2 > 3 **
Self-esteem	3.54 (0.03)	3.52 (0.03)	3.27 (0.05)	3.32 (0.06)	1 & 2 > 3 *** 1 & 2 > 4 **

Note: Covariates were controlled but not presented in Table. ^†^ *p* < 0.10. * *p* < 0.05. ** *p* < 0.01. *** *p* < 0.001.

## Data Availability

Longitudinal Study of Generation dataset is available: https://www.icpsr.umich.edu/web/DSDR/studies/22100. The 2022 survey data is currently not available.
